# Mandarin Speech Perception in Combined Electric and Acoustic Stimulation

**DOI:** 10.1371/journal.pone.0112471

**Published:** 2014-11-11

**Authors:** Yongxin Li, Guoping Zhang, John J. Galvin, Qian-Jie Fu

**Affiliations:** 1 Department of Otolaryngology, Head and Neck Surgery, Beijing TongRen Hospital, Capital Medical University, Beijing, 100730, P. R. China; 2 Department of Head and Neck Surgery, David Geffen School of Medicine, University of California Los Angeles, Los Angeles, California, United States of America; University of Salamanca- Institute for Neuroscience of Castille and Leon and Medical School, Spain

## Abstract

For deaf individuals with residual low-frequency acoustic hearing, combined use of a cochlear implant (CI) and hearing aid (HA) typically provides better speech understanding than with either device alone. Because of coarse spectral resolution, CIs do not provide fundamental frequency (F0) information that contributes to understanding of tonal languages such as Mandarin Chinese. The HA can provide good representation of F0 and, depending on the range of aided acoustic hearing, first and second formant (F1 and F2) information. In this study, Mandarin tone, vowel, and consonant recognition in quiet and noise was measured in 12 adult Mandarin-speaking bimodal listeners with the CI-only and with the CI+HA. Tone recognition was significantly better with the CI+HA in noise, but not in quiet. Vowel recognition was significantly better with the CI+HA in quiet, but not in noise. There was no significant difference in consonant recognition between the CI-only and the CI+HA in quiet or in noise. There was a wide range in bimodal benefit, with improvements often greater than 20 percentage points in some tests and conditions. The bimodal benefit was compared to CI subjects’ HA-aided pure-tone average (PTA) thresholds between 250 and 2000 Hz; subjects were divided into two groups: “better” PTA (<50 dB HL) or “poorer” PTA (>50 dB HL). The bimodal benefit differed significantly between groups only for consonant recognition. The bimodal benefit for tone recognition in quiet was significantly correlated with CI experience, suggesting that bimodal CI users learn to better combine low-frequency spectro-temporal information from acoustic hearing with temporal envelope information from electric hearing. Given the small number of subjects in this study (n = 12), further research with Chinese bimodal listeners may provide more information regarding the contribution of acoustic and electric hearing to tonal language perception.

## Introduction

For cochlear implant (CI) users with residual acoustic hearing in the contralateral ear, speech understanding may be greatly improved when the CI is combined with a hearing aid (HA). Many studies with English-speaking CI users have shown better sentence recognition in noise for combined use of CI+HA (“bimodal” listening) than with either device alone [1]–[11]. The HA provides fundamental frequency (F0) information that is not well-represented by the CI. Acoustic F0 information is especially beneficial for CI users when listening to speech in dynamic noise or competing talkers [5], [12]–[13].

Significant bimodal benefits (defined here as the difference in performance between the CI+HA and the CI alone) have been observed for monosyllabic words and phonemes [6], [9], [14]–[16]. Depending on the upper limit of aided acoustic hearing, the HA may provide useful first and second formant (F1 and F2) information for vowels and voicing information for consonants. Yoon et al. [9] found a significant bimodal benefit for perception of F1 and F2 information in vowel recognition and voicing information in consonant recognition with the CI+HA versus the CI alone, but mostly for CI subjects with “good” aided acoustic hearing (pure-tone average, or PTA <55 dB HL). Sheffield and Zeng [15] also found significantly better perception of F1 and F2 information in vowel recognition and voicing information in consonant recognition with the CI+HA compared with the CI alone. These results suggest that the bimodal benefit may be due to phonemic features besides F0 transmitted by the HA.

The amount of residual hearing has been associated with bimodal benefit in many studies. In studies where the bandwidth of available acoustic information was systematically varied, the bimodal benefit increased as the bandwidth was increased [11], [17]–[18]. Bimodal benefit has also been correlated with hearing function of the non-implanted ear. Yoon et al. [9] found that aided acoustic low-frequency pure-tone average (PTA) thresholds, calculated between 250 and 1000 Hz, were significantly correlated with the bimodal benefit for vowel recognition at low signal-to-noise ratios (SNRs). Sheffield and Zeng [15] found that vowel recognition was significantly correlated with the slope of unaided audiometric threshold functions. Zhang et al. [16] found a significant correlation between HA-aided audiometric thresholds and bimodal benefit for speech performance and quiet and in noise, as well as a significant correlation between bimodal benefit and the spectral resolution of the non-implanted ear. These studies suggest that the HA may provide useful acoustic information beyond F0, depending on audibility.

While bimodal benefits have been shown for English-speaking CI users, very few studies have examined bimodal benefits for tonal languages such as Mandarin Chinese. For tonal languages, the perception of lexical tones depends strongly on fundamental frequency (F0) cues [19]. However, the functional spectral resolution of the CI is not sufficient to support complex pitch perception needed for difficult listening tasks such as music perception, talker identification, and speech understanding in noise [20]. Efforts to improve CI users’ pitch by modifying the signal processing have shown relatively little benefit [21]–[24]. Previous studies with Mandarin-speaking CI users have shown moderately good tone recognition performance, ranging between approximately 50–80% correct [25]–[32], most likely due to access to amplitude contour and duration cues that co-vary with F0 in naturally uttered Chinese tones [29], [32]. When Mandarin-speaking CI users must rely exclusively on F0 information, tone recognition is generally poorer [30], [34]–[35].

For patients with some residual acoustic hearing, combining a HA with the CI may represent the best opportunity to improve CI users’ Chinese tone recognition. Luo and Fu [36] investigated the contribution of low frequency acoustic information to Chinese speech perception in Mandarin-speaking normal-hearing (NH) subjects listening to an acoustic simulation of bimodal listening (i.e., low frequency acoustic information presented to one ear and a CI simulation presented to the other ear). Results showed that acoustic information below 500 Hz contributed strongly to tone recognition, while acoustic information above 500 Hz contributed strongly to phoneme recognition; Chinese sentence recognition in noise improved as the bandwidth of acoustic information was increased. These results suggest that, for CI patients with residual acoustic hearing, preserving low-frequency acoustic information can improve Chinese speech recognition in noise.

While combined acoustic-electric hearing is not unusual in adult English-speaking CI patients, there are relatively few Mandarin-speaking adults who regularly use both the CI and HA. Because most of cochlear implantees in China have been children, adult CI users are less common, though those numbers will increase as the children grow older and implant criteria change. Currently, the recommended criteria for cochlear implantation for adults in China include severe-to-profound bilateral deafness, with unaided audiometric threshold PTAs (0.5–4 kHz) >80 dB HL [37]. These criteria for adults are more restrictive than in the United States, where moderate-to-severe bilateral sensorineural hearing loss is indicated. As such, it is somewhat unusual to find native adult Chinese CI users with substantial acoustic hearing in the non-implanted ear. Such patients would provide great insight into the contribution of aided acoustic hearing to Chinese CI users’ speech perception. In this study, Chinese tone, vowel, and consonant recognition in quiet and in noise were evaluated in adult Mandarin-speaking subjects who regularly used a CI and HA. Subjects were tested while listening with the CI-only, or with the CI+HA. Due to the better F0 information provided by the HA, we hypothesized that lexical tone recognition would greatly benefit from the addition of the HA to the CI. We also hypothesized that vowel recognition would be better for subjects with greater acoustic hearing range, as the HA may provide useful formant information. Finally, we hypothesized that the bimodal benefit would be greater in noise than in quiet, consistent with previous studies [3]–[4], [6]–[7], [38].

## Methods

### Subjects

Twelve Mandarin-speaking bimodal CI patients (6 male and 6 female) participated in this study. Subjects were native speakers of Mandarin Chinese and were between the ages of 16 to 24 years old. All CI subjects had more than six months of experience with their device at the time of testing. Subjects were recruited from the Department of Otolaryngology, Head and Neck Surgery of Beijing TongRen Hospital (which specifically approved this study), with no particular consideration of age at implantation, duration of profound deafness, period for bimodal experience, etiology, CI or HA type, configuration, or processor strategy. Subject demographics are shown in Table 1.

**Table 1:** Bimodal CI subject demographics.

Subject	Gender	Age	Ethology	CI exp (yrs)	Dur Deaf (yrs)
S1	F	22	LVAS	3.4	14.0
S2	F	21	Unknown	0.6	5.0
S3	F	20	Unknown	0.5	8.0
S4	M	24	LVAS	4.9	10.0
S5	M	23	Ototoxicity	3.8	12.0
S6	M	16	Unknown	2.5	5.0
S7	F	22	Unknown	6.0	16.0
S8	F	21	LVAS	0.5	21.0
S9	F	20	Unknown	2.8	17.0
S10	M	23	Unknown	4.4	19.0
S11	M	16	Unknown	2.0	14.0
S12	M	16	LVAS	1.0	13.0

CI exp = cochlear implant experience; Dur deaf = duration of deafness; F = female; M = male; LVAS = large vestibular aqueduct syndrome.

### Ethics statement

All subjects provided written informed consent prior to participating in the study, in compliance with the Institutional Review Board protocol of Beijing TongRen Hospital, Capital Medical University, China, which specifically approved this study. In terms of the minors/children enrolled in the study, the written informed consent was obtained from the next of kin, caretakers, or guardians on behalf of the minors/children enrolled in the study.

### Stimuli

Stimuli for tone, consonant, and vowel recognition tests were drawn from the Chinese Standard Database [39]. For Chinese tone recognition, two male and two female speakers each produced four tones for the four Mandarin Chinese monosyllabic words “ba”, “bi”, “bu”, “bo”, resulting in a total of 64 tone tokens. For Chinese vowel recognition, two male and two female speakers each produced sixteen Mandarin Chinese monosyllabic words in a/d/-vowel context with tone 1 (/a/,/i/,/u/,/ai/,/ao/,/ou/,/iao/,/iu/,/uo/,/ui/,/ang/,/eng/,/ong/,/ing/,/uan/, and/un/), resulting in a total of 64 vowel tokens. Key acoustic features of vowel stimuli are shown in Table 2. For Chinese consonant recognition, two male and two female speakers each produced tone 1 for/a/in a consonant-/a/context, for the 20 Mandarin Chinese initial consonants (/b/,/p/,/m/,/f/,/d/,/t/,/l/,/g/,/k/,/h/,/j/,/q/,/x/,/zh/,/ch/,/sh/,/z/,/c/,/s/and/w/), resulting in a total of 80 consonant tokens.

**Table 2:** Production-based acoustic features for vowel stimuli.

	F0 (Hz)			F1 (Hz)			F2 (Hz)		
Talker	Mean	Min	Max	Mean	Min	Max	Mean	Min	Max
f1	285	267	297	582	299	979	1707	867	2827
f2	278	268	291	600	295	1116	1636	767	2932
f AVE	282	268	294	591	297	1048	1672	817	2880
m1	165	135	187	566	309	857	1670	906	2427
m2	177	163	188	589	303	897	1777	949	2454
m AVE	171	149	188	578	306	877	1724	928	2441
AVE	228	212	241	587	301	967	1711	878	2663

f = female; m = male; F0 = fundamental frequency; F1 = first formant; F2 = second formant.

### Procedure

Closed-set identification tasks were used to measure Chinese tone recognition (4-alternative, forced-choice, or 4AFC), vowel recognition (16AFC), and consonant recognition (20AFC). For each trial within a given test, a stimulus was randomly selected from the token list without repetition and presented to the subject, who responded by clicking on one of the response choices shown on screen. For tone recognition, the 4 response choices were labeled “Tone 1”, “Tone 2”, “Tone 3”, and “Tone 4”. For vowel recognition, each of the 16 response choices was labeled in a d/V/context using Pinyin symbol and the corresponding Chinese character. For the consonant recognition, each of the 20 response choices was labeled in a C/a/using Pinyin symbol and the corresponding Chinese character. Responses were collected by the experimental software (Mandarin i-CAST software developed by Qian-Jie Fu and freely available at http://icast.emilyfufoundation.org) and scored in terms of percent correct. No trial-by-trial feedback or training was provided. Because of time constraints (data were collected following a routine clinical appointment) and the number of conditions and tests [2 listening (CI, CI+HA)×2 noise (quiet, noise)×3 tests (tone, vowel, consonant) = 12], only one run was collected for each test and listening condition. The total amount of time required to complete all tests was 2–3 hours.

Chinese tone, vowel, and consonant recognition were measured in quiet and in noise under two listening conditions: CI alone and combined CI+HA. Because of time constraints and subject availability, the HA alone condition was not tested. For the CI alone condition, the HA was removed but the HA ear was not plugged. The two listening conditions were evaluated in random order for each subject. For testing in noise, speech-weighed steady noise (1000-Hz cutoff frequency, −12 dB/octave) was used. The SNR was fixed at +5 dB, and was calculated in terms of the long-term root-mean square (RMS) of the speech signal and noise. The onset and offset of the noise was 500 ms before the target speech token. Speech and noise were mixed at the target SNR of +5 dB, and the combined signal and noise was then scaled to the output (65 dBA). Testing was conducted in a sound-treated booth. Subjects were seated directly facing a single loudspeaker 1 m away. CI subjects were tested with their clinical CI and HA settings; these settings were not changed during testing.

## Results

**Figure 1:**
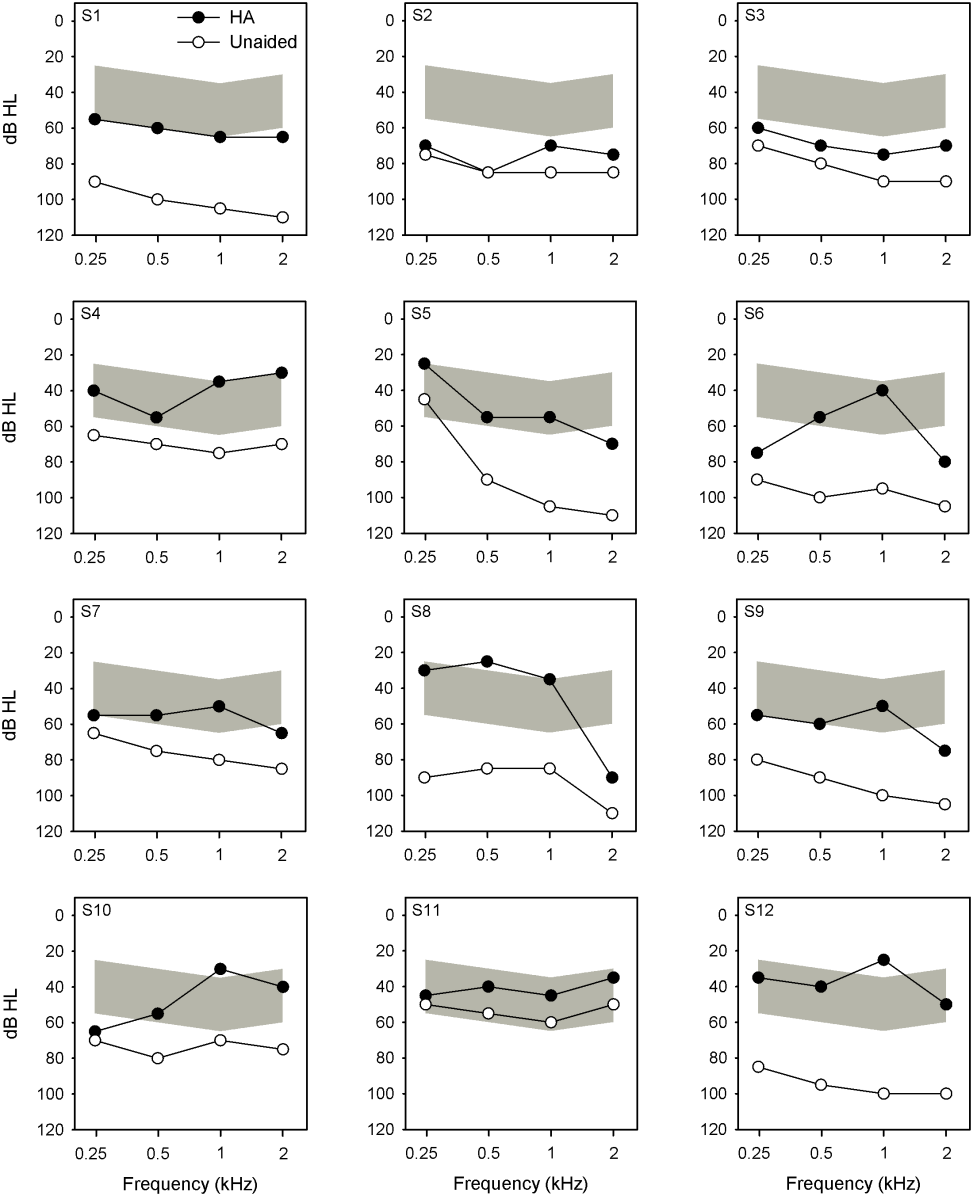
Aided and unaided thresholds for bimodal subjects. The x-axis shows audiometric frequency and the y-axis shows threshold in dB HL. The open circles show unaided threshold and the filled circles show HA-aided thresholds. The shaded area shows the range of hearing levels for conversational speech levels (i.e., the “speech banana”). HA = hearing aid.

Figure 1 shows CI subjects’ unaided (white symbols) and HA-aided (black symbols) audiometric thresholds at 0.25, 0.5, 1, 2, and 4 kHz. Audiometry was conducted in sound field using warble tones. Unaided pure-tone average (PTA) thresholds between 0.25 and 2 kHz ranged from 54 to 101 dB HL; with the exception of subject S11, unaided thresholds were above the range of conversational speech levels (gray shaded areas in each panel). HA-aided PTA thresholds ranged from 38 to 75 dB HL; in many cases, the HA amplification boosted thresholds within the range of conversational speech levels. With the CI-only, PTA thresholds ranged from 24 to 48 dB HL. With the CI+HA, PTA thresholds ranged from 23 to 41 dB HL. With the CI-only or with the CI+HA, thresholds were well within the range of conversational speech levels. Note that no “real-ear” measurements were conducted to confirm sound pressure levels in the HA ear.

**Figure 2:**
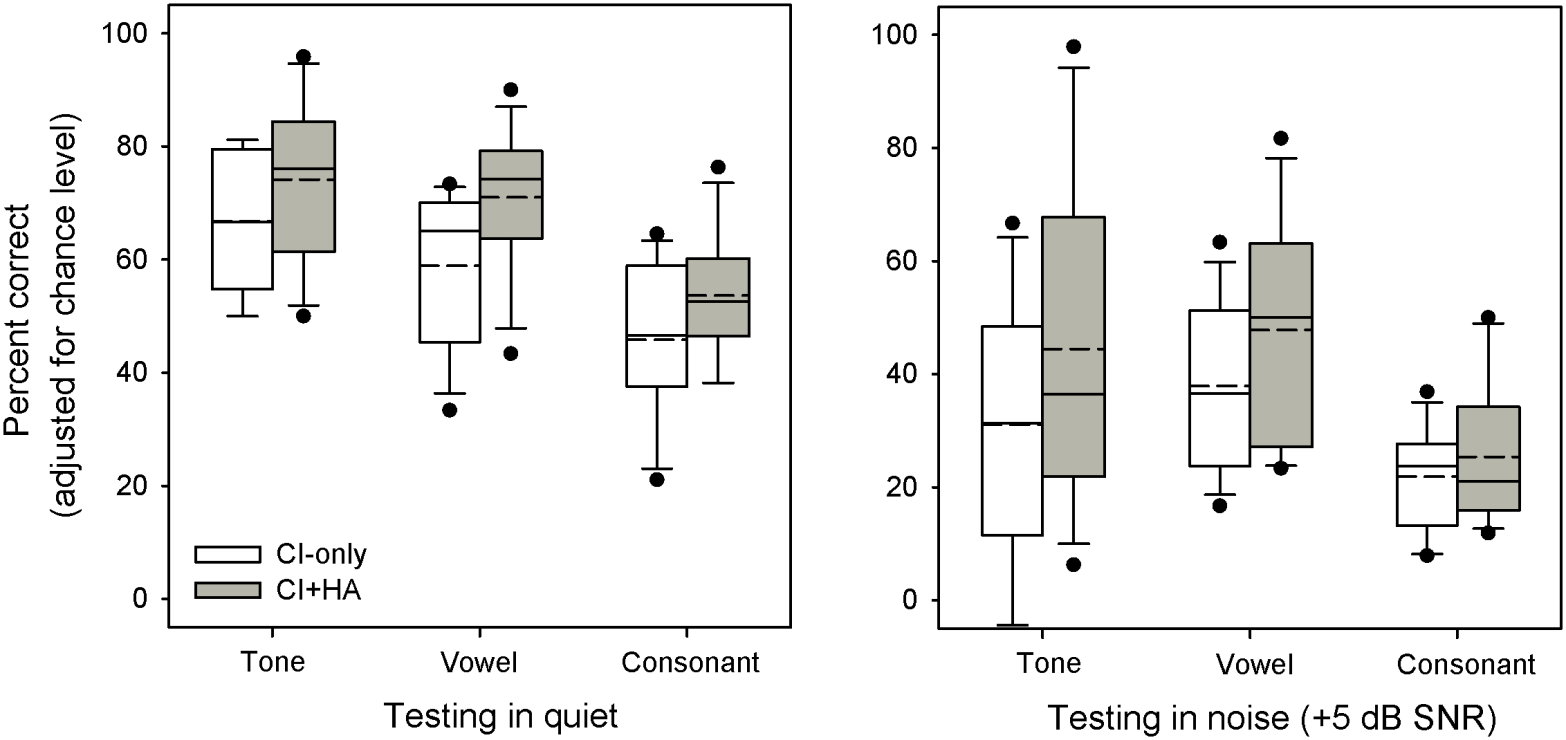
Boxplots for speech performance with CI-only or with CI+HA. The left panel shows performance measured in quiet and the right panel shows performance measured in noise. The white boxes show CI-only performance and the gray boxes show CI+HA performance. The boxes show the 25th and 75th percentiles, the short dashed lines show median value, the solid lines show mean value, the error bars show the 10th and 90th percentiles, and the circles show outliers. CI = cochlear implant; HA = hearing aid.

Figure 2 shows boxplots of tone, vowel, and consonant recognition in quiet and in noise, with the CI only or with the CI+HA. All scores were corrected for chance level performance (25% for tones, 6.25% for vowels, and 5% for consonants).

Relative to the CI-only, mean tone recognition with the CI+HA improved from 66.8% to 74.1% correct in quiet (+7.3 points), and from 31.1% to 44.5% correct in noise (+13.4 points). A two-way repeated measures of variance (RM ANOVA), with listening condition (CI-only, CI+HA) and SNR (quiet, 5 dB) as factors, showed that tone recognition was significantly affected by listening condition [F(1,11) = 16.3, p = 0.002] and SNR [F(1,11) = 40.0, p<0.001]; there were no significant interactions. Post-hoc Bonferroni pairwise comparisons showed that performance was significantly better in quiet than in noise for both the CI-only and CI+HA listening conditions (p<0.05 in both cases). Post-hoc Bonferroni pairwise comparisons also showed that tone recognition was significantly better with the CI+HA in noise (p<0.05), but not in quiet (p>0.05).

Relative to the CI-only, mean vowel recognition with the CI+HA improved from 58.9% to 71.0% correct in quiet (+12.1 points), and from 37.9% to 47.8% correct in noise (+9.9 points). A two-way RM ANOVA, with listening condition and SNR as factors, showed that vowel recognition was significantly affected by listening condition [F(1,11) = 10.9, p = 0.007] and SNR [F(1,11) = 42.5, p<0.001]; there were no significant interactions. Post-hoc Bonferroni pairwise comparisons showed that performance was significantly better in quiet than in noise for both the CI-only and CI+HA listening conditions (p<0.05 in both cases). Different from tone recognition, post-hoc Bonferroni pairwise comparisons showed that vowel recognition was significantly better with the CI+HA in quiet (p<0.05), but not in noise (p>0.05).

Relative to the CI-only, mean consonant recognition with the CI+HA improved from 45.8% to 53.6% correct in quiet (+7.8 points), and from 21.9% to 25.84% correct in noise (+3.5 points). Note that subject S12 was unable to complete the consonant recognition tests. A two-way RM ANOVA, with listening condition and SNR as factors, showed that consonant recognition was significantly affected by listening condition [F(1,10) = 6.15, p = 0.033] and SNR [F(1,10) = 88.6, p<0.001]; there were no significant interactions. Post-hoc Bonferroni pairwise comparisons showed that performance was significantly better in quiet than in noise for both the CI-only and CI+HA listening conditions (p<0.05 in both cases). However, post-hoc Bonferroni pairwise comparisons showed no significant difference in consonant recognition between the CI-only and CI+HA listening conditions in quiet or in noise (p>0.05 in both cases).

**Figure 3:**
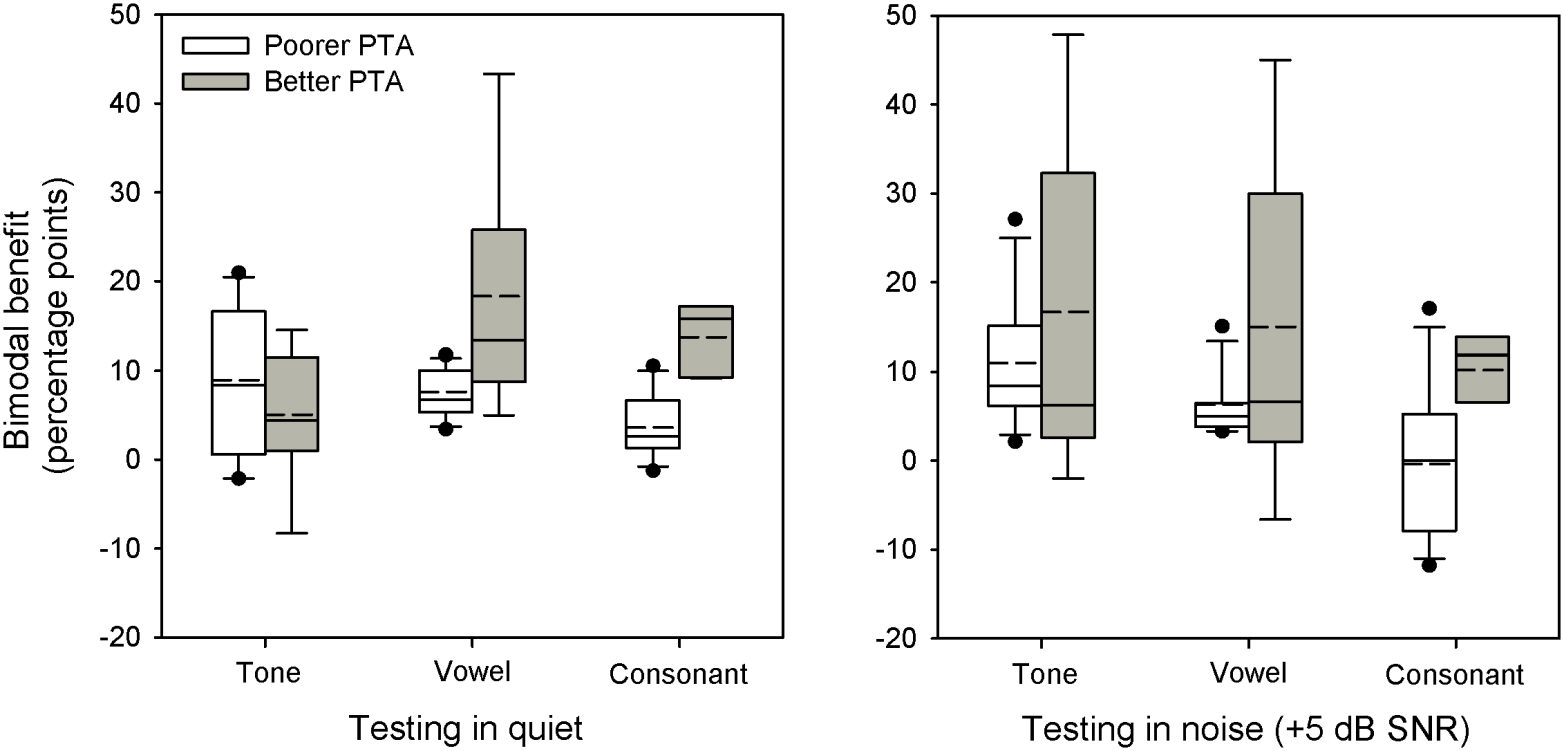
Boxplots for bimodal benefit. The left panel shows performance measured in quiet and the right panel shows performance measured in noise. The white boxes show performance for the CI group with poorer HA-aided PTA thresholds (>50 dB HL for audiometric frequencies between 0.25 and 2 kHz) for the CI group with better HA-aided PTA thresholds (<50 dB HL) The boxes show the 25th and 75th percentiles, the short dashed lines show median value, the solid lines show mean value, the error bars show the 10th and 90th percentiles, and the circles show outliers. CI = cochlear implant; HA = hearing aid; PTA = pure-tone average.

Paired to t-tests showed no significant difference in bimodal benefit (CI+HA–CI-only) between quiet and noise for tones (p = 0.201), vowels (p = 0.332), or consonants (p = 0.107). Figure 3 shows boxplots of the bimodal benefit for tones, vowels, and consonants in quiet and in noise; data are grouped according to CI subjects with “better” (<50 dB HL; n = 5) or “poorer” (>50 dB HL; n = 7) HA-aided PTA thresholds averaged across 0.25, 0.5, 1, and 2 kHz. The frequency range for PTAs was similar as used by Wang et al. [35] when measuring residual acoustic hearing. The bimodal benefit was calculated using the scores corrected for chance level.

For tone recognition, the mean bimodal benefit in quiet was 9.1 percentage points (range: −2.1–20.9) for the poorer group and 5.1 points (range: −8.3–14.5) for the better group. In noise, the mean bimodal benefit in quiet was 11.0 points (range: 2.1–27.1) for the poorer group and 16.7 points (range: 5.1–45.1) for the better group. A split-plot RM ANOVA, with SNR (quiet, 5 dB) as the within subject factor and HA-aided threshold (poorer, better) as the between subject factor, showed no significant effects for SNR [F(1,10) = 1.14, p = 0.311, observed power = 0.17] or HA-aided threshold [F(1,10) = 0.03, p = 0.874, observed power = 0.16].

For vowel recognition, the mean bimodal benefit in quiet was 7.6 points (range: 3.4–11.7) for the poorer group and 18.3 points (range: 5.0–43.3) for the better group. In noise, the mean bimodal benefit in quiet was 6.3 points (range: 3.3–15.0) for the poorer group and 15.0 points (range: −6.6–45.0) for the better group. A split-plot RM ANOVA, with SNR as the within-subject factor and HA-aided threshold as the between-subject factor, showed no significant effects for SNR [[F(1,10) = 0.2, p = 0.665, observed power = 0.07] or HA-aided threshold [F(1,10) = 2.29, p = 0.161, observed power = 0.28].

For consonant recognition, the mean bimodal benefit in quiet was 3.6 points (range: −1.3–10.5) for the poorer group and 13.7 points (range: 9.2–17.2) for the better group. In noise, the mean bimodal benefit in quiet was −0.4 points (range: −11.8–17.1) for the poorer group and 10.2 points (range: 2.6–14.5) for the better group. A split-plot RM ANOVA, with SNR as the within-subject factor and HA-aided threshold as the between-subject factor, showed no significant effect for SNR [F(1,9) = 0.02, p = 0.994, observed power = 0.05], but a significant effect for HA-aided threshold [F(1,9) = 10.7, p = 0.010, observed power = 0.82].

Key demographic variables were compared to CI-only performance, CI+HA performance, and bimodal benefit; scores were corrected for chance level performance. As all subjects were implanted at 16 yrs or later, only duration of deafness and CI listening experience were used as demographic variables. The significance level was adjusted to correct for family-wise error (Bonferroni-adjusted p<0.003). A significant was found only between CI experience and bimodal benefit for tone recognition in quiet (r = 0.79; p = 0.003). There were no significant correlations between CI experience and CI-only or CI+HA performance for any of the speech tests in quiet or in noise, and there were no significant correlations between CI experience and bimodal benefit for vowel or consonant recognition in quiet or in noise, or for tone recognition in noise. There were no significant correlations between duration of deafness and CI-only performance, CI+HA performance, or bimodal benefit for any of the speech tests.

## Discussion

The present results showed a clear bimodal benefit for tone, consonant, and vowel recognition in quiet, and for tone and vowel recognition in noise. The bimodal benefit appeared to be greater for CI subjects with better HA-aided PTA thresholds, especially for consonant recognition. Greater CI experience was associated with greater bimodal benefit for tone recognition in quiet. Below, we discuss the results in greater detail.

### Bimodal benefit in quiet

Given the dependence on F0 cues for tone recognition, one might have expected that the greatest bimodal benefit would have been for tones, as the addition of the HA would have provided low-frequency pitch cues. For the present tone stimuli, amplitude contour and duration cues were available, as stimuli were natural productions of Chinese tones. These cues are known to co-vary with F0 [19], [33], [40]–[41]. It is possible that the availability of amplitude and duration cues may have weakened the dependence on F0 cues and therefore, the bimodal benefit relative to vowels and consonants. Note that for some subjects, the bimodal benefit for tones was substantial (18.7 and 20.9 points, after correcting for chance level, for subjects S5 and S7, respectively). With the CI alone, only 33.3% of subjects scored 75% percent correct or better (after correcting for chance level performance); with the CI+HA, 50% of subjects scored 75% percent correct or better. This suggests that the addition of the HA to the CI was key to good tone recognition performance for many of the present CI subjects.

Similarly, one might expect that the bimodal benefit would have been weakest for consonants, as high-frequency information would have been largely represented by the CI. However, the mean bimodal benefit was similar for tones and consonants. Again, some subjects experienced substantial bimodal benefit (15.8, 17.2, and 17.2 points for subjects S4, S8, and S11, respectively, after correcting for chance level performance). The addition of the HA may have strengthened voice cues and may have provided better coding of aperiodic consonant information.

The greatest bimodal benefit in quiet was observed for vowels. Again, some subjects experienced substantial bimodal benefit (20.0 and 43.1 points for subjects S8 and S4, respectively, after correcting for chance level performance). While F0 information is not required for vowel recognition, the HA may have provided better representation of F1 (which ranged from 301 to 967 Hz for the present stimuli) and even F2 information (which ranged from 878 to 2663 Hz), depending on the audibility of aided acoustic hearing.

### Bimodal benefit in noise

Mean speech performance was significantly poorer in noise than that in quiet for all speech measures and listening conditions. Interestingly, a significant bimodal benefit was observed for tone recognition in noise, but not in quiet. The bimodal benefit for tone recognition in noise was substantial for some subjects (27.1, 27.1, and 47.9 points, after correcting for chance level performance, for subjects S6, S8, and S4, respectively). The bimodal advantage for tone recognition in noise is consistent with previous studies that show a bimodal advantage for sentence recognition in noise [5], [12]–[13]. However, some subjects experienced nearly no benefit or even a deficit with the CI+HA, relative to the CI alone (2.1 and −2.0 points, after correcting for chance level performance, for subjects S3 and S11, respectively). Different from testing in quiet, there was no significant bimodal benefit for vowel recognition in noise. Again, the bimodal benefit for vowel recognition in noise was substantial for some subjects (15.0, 25.0, and 45.0 points, after correcting for chance level performance, for subjects S7, S8, and S4, respectively).

The lack of bimodal benefit for consonants in noise may have been due to the relatively low SNR (+5 dB), which was calculated according to the long-term RMS of the noise and the speech token. For consonants, most of the energy in the speech token was for the vowel portion of the C/a stimuli which would have contributed strongly to the estimate of the long-term RMS. As such, the consonant portion may have been masked by the noise. Even so, some subjects received a substantial bimodal benefit (13.2, 14.5, and 17.1 points, after correcting for chance level performance, for subjects S4, S10, and S7, respectively). However, some subjects also received a substantial bimodal deficit (−7.8 and −11.8 points, after correcting for chance level performance, for subjects S1 and S7, respectively). It is unclear whether such large bimodal deficits are due to sub-optimal HA settings or to other factors.

Previous sentence recognition studies with English-speaking CI users have shown that the bimodal benefit was generally greater in noise than in quiet [3]–[4], [6]–[7]. Previous phoneme recognition studies with English-speaking bimodal CI users have also shown that the bimodal benefit was generally small in quiet and greater in noise. Kong and Braida [14] found no significant difference in vowel or consonant recognition in quiet between the CI-only and CI+HA. Sheffield and Zeng [15] found no significant difference in overall vowel recognition in quiet between the CI-only and CI+HA; however, overall vowel recognition in noise and information transfer of F1 and F2 information was significantly better with the CI+HA than with the CI alone. In this study, a significant bimodal benefit for vowel recognition was observed in quiet, but not in noise. In Sheffield and Zeng [15], consonant recognition was significantly better with the CI+HA than with the CI alone in quiet, but not in noise. In this study, there was no significant bimodal benefit for consonant recognition in quiet or in noise. Differences in speech materials, testing methods, and subject groups may explain some of the inconsistencies between the present and previous studies.

Improved perception of F0 information with the HA may allow bimodal CI users to better segregate speech from noise, typically measured using sentence materials. In this study, the bimodal benefit was assessed for lexical tones, vowels and consonants. It is possible that the lack of contextual cues focused listeners’ attention to other cues afforded by the HA besides F0, such as F1 and F2 information, depending on the amount of residual aided acoustic hearing. Averaged across all talkers and vowel stimuli, the mean range of F1 was 301–967 Hz and the mean range of F2 was 878–2663 Hz. F1 and F2 cues may have been in the audible range with the HA for some subjects (see Fig. 1). As such, the HA provides important acoustic features for phoneme recognition that is beneficial in both quiet and noise.

### Dependence of bimodal benefit on aided acoustic hearing

The bimodal benefit for consonant recognition appeared to depend on the audibility of acoustic information provided by the HA, coarsely divided in this study according to whether PTA thresholds were above or below 50 dB HL (see Fig. 3). This result is consistent with previous speech recognition studies that have shown that the bimodal benefit depends on the amount of residual acoustic hearing [9], [15]–[16], [42]. For example, Yoon et al. [9] found that the CI subjects with “better” HA-aided PTA thresholds (<55 dB HL across audiometric frequencies 0.25, 0.5, 0.75, and 1 kHz) exhibited a clear bimodal benefit for vowel and sentence recognition in noise, while CI subjects with “poorer” PTA thresholds (>55 dB) received little bimodal benefit across speech tests and SNRs. In this study, PTA thresholds were averaged across 0.25, 0.5, 1, and 2 kHz, and the breakpoint between “better” and “poorer” HA-aided thresholds was 50 dB HL.

For tone and vowel recognition, there was no significant relationship between HA-aided thresholds and bimodal benefit. Note that statistical power was quite low for these analyses, given the limited number of subjects. With the exception of tone recognition in quiet, the mean bimodal benefit was greater for subjects with better HA-aided thresholds for nearly all tests in quiet and in noise. Besides the limited number of subjects, the lack of statistical significance may be due to adequate audibility for tone and vowel cues even when HA-aided thresholds were greater than 50 dB HL.

It is important to note that PTA thresholds reported and analyzed in this study provide only partial information regarding the audibility of signals with aided acoustic hearing. In this study, the effects of HA compression and signal processing were unknown, and there was no verification of sound pressure level at the ear canal, as is common in clinical practice. HA prescription has been also shown to greatly affect CI users’ bimodal benefit [43]. The present subjects were all tested using their clinically assigned HAs and CIs, presumably set for conversational speech; these settings were not changed during testing. Further testing with a greater number of Chinese bimodal CI users, as well as greater control of HA variables (e.g., threshold, compression, etc.) may provide greater insight into the role of aided acoustic hearing levels on bimodal benefit. Such information is needed to optimize HAs for optimal use with contralateral CIs.

### Demographic factors

Only the bimodal benefit for tone recognition in quiet was correlated with CI experience; no other demographic variables were correlated with CI-only performance, CI+HA performance, or bimodal benefit for any of the remaining speech measures in quiet or in noise. Subjects in the present study were all adults, late-implanted with long-term experience of acoustic hearing, whether aided or unaided. As such, the relatively late onset of auditory deprivation may not be comparable to that of pre-lingually deafened CI users. In studies with pediatric Mandarin-speaking CI users, age at implantation has been negatively correlated with speech performance [28], [44]–[45] In these studies, the duration of deafness was generally calculated between birth and age at implantation, and speech development worsens as the auditory deprivation increases. With the present adults, the auditory deprivation may have occurred after sufficient development of speech patterns via acoustic hearing.

Unfortunately, little information was available regarding the extent of acoustic hearing (aided or unaided) or unaided for the present subjects. As such, the correlation with duration of deafness may not reflect age-accurate diagnoses of onset of sever-to-profound deafness. Although CI experience was not significantly correlated with speech scores in quiet or in noise, it was significantly correlated with the bimodal benefit for tone recognition in quiet. This suggests that CI users must learn to combine acoustic and electric stimulation for tone recognition after implantation. As mentioned earlier, the perception of lexical tones depends strongly F0 cues [19] in acoustic hearing. While CIs do not provide strong F0 cues due to coarse spectral resolution, CI users are able to perceive some tonal information by using temporal cues such as periodicity-related amplitude fluctuations and similarities between the fundamental F0 contour and the amplitude envelope [23], [33]. The correlation between CI experience and bimodal benefit for lexical tones suggests that CI patients are able to learn to combine fine structure cues via acoustic hearing with temporal envelope cues via electric hearing. Combining these acoustic and electric cues may require longer bimodal listening experience or even explicit training.

### Implications for Mandarin-speaking CI users

Because of the current restrictive criteria (severe-to-profound bilateral deafness) for cochlear implantation in adult Chinese individuals, it is difficult to find large numbers of adult, post-lingually deafened bimodal listeners in China. This difficulty contributed to the small sample size (n = 12) of the present study. However, the present data suggest that residual acoustic hearing in the non-implanted ear can improve many aspects of Mandarin speech perception by CI users. As such, less restrictive criteria for adults, as found in the United States (moderate-to severe bilateral deafness), may allow for some contribution of aided acoustic hearing to electric hearing, thereby providing a better overall benefit for cochlear implantation. As the number of Chinese bimodal listeners increases, further research with more patients may provide additional information regarding the benefit of combined acoustic and electric hearing for tonal language perception.

There was a clear benefit for combined use of a CI and HA for most speech measures. If possible, residual acoustic hearing should be combined with electric hearing to maximize the benefit of cochlear implantation. The correlation between bimodal benefit and CI experience for tone recognition suggests that bimodal CI users should wear both devices as much as possible to learn to combine acoustic and electric hearing. Alternatively, bimodal training may accelerate this learning process. With English-speaking CI users, Zhang et al. [16] found significant improvements in bimodal speech performance after bimodal training on home computers, although the benefit was largely due to improved CI-only performance.

Combined with findings from previous studies showing that increased bandwidth for acoustic hearing is associated with better bimodal performance [11], [17]–[18], efforts should be made to implant patients with substantial aided acoustic hearing. Alternatively, HA signal processing may be modified (“frequency transposition”) to better preserve formant frequency cues as well as F0. It is unclear if how frequency transposition in acoustic hearing may interact with the apical frequency mismatch associated with electric hearing. If HA signal processing is optimized for use with a CI, bimodal listening experience and/or training may help Mandarin-speaking CI users to better combine speech cues from acoustic and electric hearing.

## Conclusions

Chinese tone, vowel and consonant recognition was measured in 12 adult Mandarin-speaking subjects who regularly used a HA with their CI; speech performance was measured with the CI-only and with the CI+HA. Major findings include:

1. Performance was significantly better with the CI+HA than with the CI alone for tone recognition in noise and vowel recognition in quiet. There was no significant difference in consonant recognition between the CI+HA and the CI alone in quiet or in noise.
2. There was no significant difference in bimodal benefit (CI+HA–CI alone) between quiet and noise for tones, vowels, or consonants. In quiet, the bimodal benefit was greatest for vowels, suggesting that the HA provided important formant information beyond F0. In noise, the bimodal benefit was similar for tones and vowels, with no benefit for consonants.
3. When CI subjects were group according to “better” (<50 dB HL) and “poorer” (>50 dB HL) HA-aided PTA thresholds, the mean bimodal benefit was generally greater for subjects with better HA-aided thresholds. However, a significant difference between these two groups was only observed in consonant recognition, possibly because of improved audibility of voicing cues.
4. Among demographic factors, only CI experience was significantly correlated with the bimodal benefit for tone recognition in quiet, suggesting that bimodal CI users must learn to combine fine structure cues from acoustic hearing with temporal envelope cues from electric stimulation.
